# Neuron-derived extracellular vesicles in blood reveal effects of exercise in Alzheimer’s disease

**DOI:** 10.1186/s13195-023-01303-9

**Published:** 2023-09-20

**Authors:** Francheska Delgado-Peraza, Carlos Nogueras-Ortiz, Anja Hviid Simonsen, De’Larrian DeAnté Knight, Pamela J. Yao, Edward J. Goetzl, Camilla Steen Jensen, Peter Høgh, Hanne Gottrup, Karsten Vestergaard, Steen Gregers Hasselbalch, Dimitrios Kapogiannis

**Affiliations:** 1 Laboratory of Clinical Investigation, Intramural Research Program, National Institute On Aging, National Institutes of Health, Baltimore, MD 21224 USA; 2 Danish Dementia Research Centre, Copenhagen University Hospital - Rigshospitalet, 2100 Copenhagen, Denmark; 3 Department of Medicine, University of California San Francisco, San Francisco, CA 94143 USA; 4 Research Department, Campus for Jewish Living, San Francisco, CA 94112 USA; 5 Department of Neurology, Zealand University Hospital, 4000 Roskilde, Denmark; 6 Department of Clinical Medicine, University of Copenhagen, 1165 Copenhagen, Denmark; 7 Department of Neurology, Dementia Clinic, Aarhus University Hospital, 8200 Aarhus, Denmark; 8 Department of Neurology, Dementia Clinic, Aalborg University Hospital, 9000 Aalborg, Denmark

**Keywords:** Extracellular vesicles, Biomarkers, Alzheimer’s disease, proBDNF, BDNF, Humanin, ADEX, APOE, Exercise, Exerkines

## Abstract

**Background:**

Neuron-derived extracellular vesicles (NDEVs) in blood may be used to derive biomarkers for the effects of exercise in Alzheimer’s disease (AD). For this purpose, we studied changes in neuroprotective proteins proBDNF, BDNF, and humanin in plasma NDEVs from patients with mild to moderate AD participating in the randomized controlled trial (RCT) of exercise ADEX.

**Methods:**

proBDNF, BDNF, and humanin were quantified in NDEVs immunocaptured from the plasma of 95 ADEX participants, randomized into exercise and control groups, and collected at baseline and 16 weeks. Exploratorily, we also quantified NDEV levels of putative exerkines known to respond to exercise in peripheral tissues.

**Results:**

NDEV levels of proBDNF, BDNF, and humanin increased in the exercise group, especially in APOE ε4 carriers, but remained unchanged in the control group. Inter-correlations between NDEV biomarkers observed at baseline were maintained after exercise. NDEV levels of putative exerkines remained unchanged.

**Conclusions:**

Findings suggest that the cognitive benefits of exercise could be mediated by the upregulation of neuroprotective factors in NDEVs. Additionally, our results indicate that AD subjects carrying APOE ε4 are more responsive to the neuroprotective effects of physical activity. Unchanged NDEV levels of putative exerkines after physical activity imply that exercise engages different pathways in neurons and peripheral tissues. Future studies should aim to expand upon the effects of exercise duration, intensity, and type in NDEVs from patients with early AD and additional neurodegenerative disorders.

**Trial registration:**

The Effect of Physical Exercise in Alzheimer Patients (ADEX) was registered in ClinicalTrials.gov on April 30, 2012 with the identifier NCT01681602.

**Graphical abstract:**

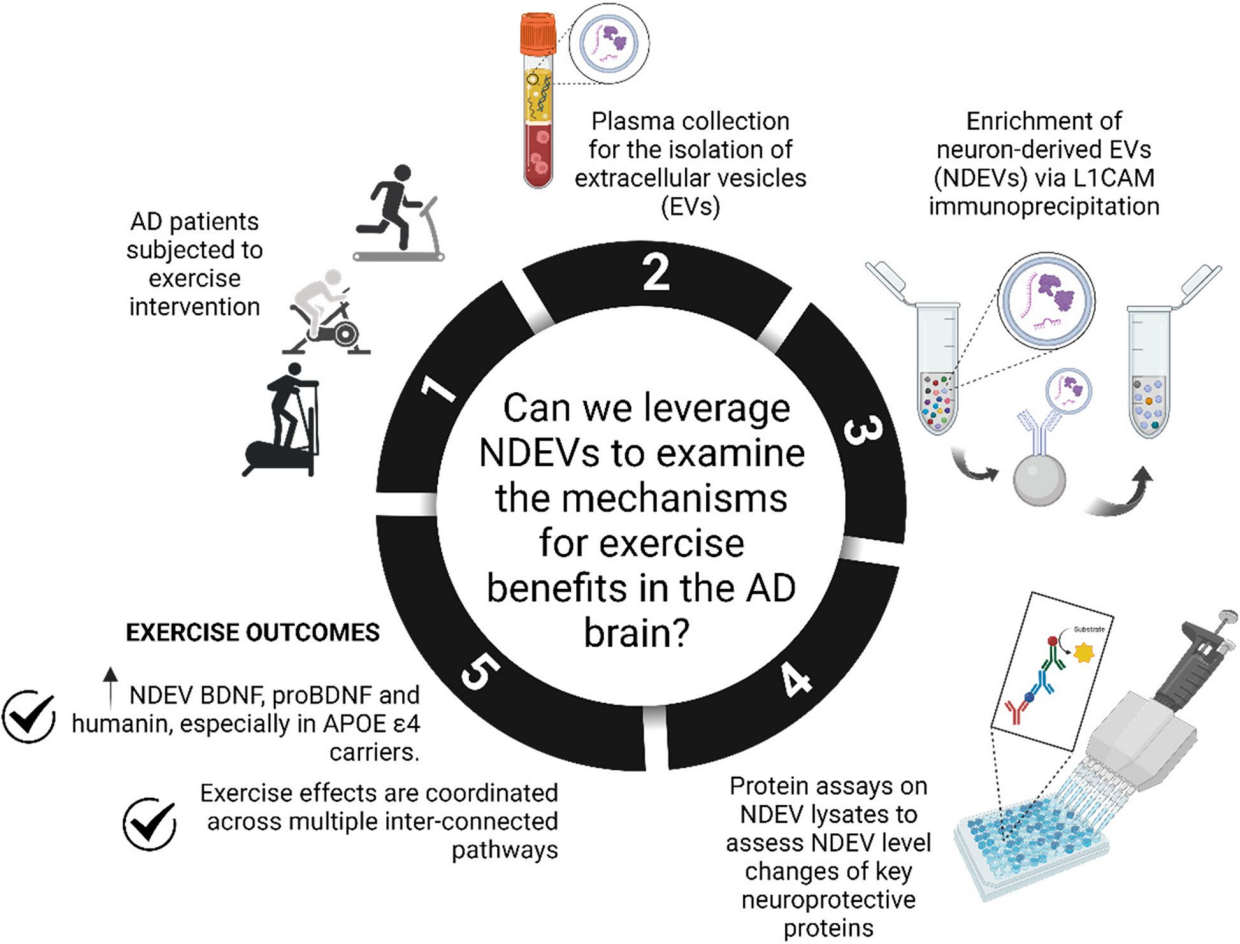

**Supplementary Information:**

The online version contains supplementary material available at 10.1186/s13195-023-01303-9.

## Background

Epidemiological studies indicate that lifestyle factors, such as a healthy diet and physical activity, may reduce the incidence of Alzheimer’s disease (AD) [1]. Multiple clinical trials assessing exercise as a non-pharmacological intervention for AD have found improvements in cognition, neuropsychiatric symptoms, and physical function. In elderly people with increased familial and genetic risk of AD, physical activity by itself or in conjunction with diet and cognitive training can improve or maintain cognitive and physical functions [2, 3]. The randomized controlled trial (RCT) ADEX [short for “Preserving Cognition, Quality of Life, Physical Health and Functional Ability in Alzheimer’s Disease (AD): The Effect of Physical Exercise”] evaluated the cognitive [4, 5] and functional [6] effects of exercise in community-dwelling individuals with mild to moderate AD [7]. In ADEX, 16 weeks of moderate to high-intensity aerobic exercise had a positive effect on neuropsychiatric symptoms [4, 8]. Moreover, the primary outcome, the symbol digit modalities test (SDMT), showed a lesser cognitive decline in participants achieving high exercise attendance and training intensity compared to control participants [4], suggesting a positive effect of relatively intense exercise on cognition.

To unravel the molecular mechanisms underlying ameliorative effects of exercise in AD, studies utilizing the ADEX cohort assessed biomarkers for classic AD pathologies [9], neurodegeneration [10, 11], and neuroinflammation [12] in cerebrospinal fluid (CSF), plasma, and serum. Results did not reveal any exercise effects on CSF Aβ, tau, synaptic proteins, or serum neurofilament light chain (NfL), suggesting that the positive functional and cognitive effects observed were perhaps attributable to activation of neuroprotective and neuroregenerative mechanisms rather than the main pathogenic cascades.

Exercise can modulate brain function both directly, through increased blood flow and cellular respiration, and indirectly, through the release of exercise-induced cytokines, termed “exerkines”, that operate in an endocrine-like manner [13]. Among neuronal proteins known to be induced by exercise are brain-derived neurotrophic factor (BDNF) and humanin, neuroprotective agents with recognized therapeutic potential in AD. Both molecules regulate synapse development and plasticity and promote neuronal survival in the face of noxious stimuli, and their depletion is part of AD pathogenesis [14].

To gain a window into the molecular changes induced by exercise in neurons, we leveraged circulating neuron-derived extracellular vesicles (NDEVs). Extracellular vesicles (EVs) are lipid membrane-enclosed nanoparticles secreted by all cells and present in plasma. These nanoparticles carry variable cargo and play critical roles in inter-cellular communication [13]. EVs secreted by brain neurons can cross the blood–brain barrier (BBB) and are detected in blood [15], thus representing a window into the brain. We and others have isolated NDEVs via immunoprecipitation targeting the neuronal cell-adhesion molecule L1CAM and have shown that NDEVs can be leveraged as a diagnostic tool for AD and other neurological disorders [16–19] and as surrogates of target engagement in clinical trials [20], including trials in AD [21]. Therefore, NDEVs reflect the state of signaling pathways in neurons and can help interrogate mechanisms engaged by pharmacologic and non-pharmacological interventions.

In this study, we hypothesized that neuronal effects of exercise may be reflected by increased NDEV levels of proBDNF, BDNF, and humanin and tested this hypothesis utilizing plasma samples from ADEX. To further explore whether putative exerkines mediate neuronal effects of exercise, we quantified NDEV levels of several candidates, especially irisin, and studied their associations with proBDNF, BDNF, and humanin.

## Methods

A description of the ADEX cohort, EV characterization methodology, and statistical analyses employed are included in Supporting Information.

### Plasma samples from the ADEX cohort

We studied all remaining 162 plasma samples from the ADEX cohort, sourced from 47 participants in the control group (*n* = 38 at baseline, *n* = 43 at 16 weeks), and 48 participants in the exercise group (*n* = 44 at baseline, *n* = 37 at 16 weeks).

### NDEV isolation

Plasma was processed in accordance with guidelines for pre-analytical factors for EV isolation and biomarker analysis [22, 23]. Blood was collected in EDTA polypropylene tubes and within 1 h centrifuged at 3000 rpm for 10 min at 4 °C. Supernatant plasma was divided into 250 µL aliquots and stored in a central biobank at − 80 °C. Plasma aliquots were received and processed blindly by investigators at the National Institute on Aging. NDEVs were isolated following an immunoaffinity capture methodology extensively characterized by us [17–20] and others [24, 25] targeting L1 cell adhesion molecule (L1CAM), a transmembrane neuronal protein sorted to EVs. Briefly, 250 µL of plasma was defibrinated via incubation with 100 µL of Pacific Hemostasis Thromboplastin-DS (cat. no. 100354; Thermo Fisher Scientific) for 45 min at room temperature (RT), followed by dilution with 150 µL of Dulbecco’s phosphate-buffered saline (DPBS) -1X supplemented with 1X protease (cOmpleteTM Protease Inhibitor Cocktail; cat no. 04693116001; Roche) and phosphatase inhibitors (Halt™ Phosphatase Inhibitor Cocktail; cat no.78427; Thermo Fisher Scientific), and sedimentation at 3000 × g for 15 min at RT. The supernatant was transferred to a sterile 1.5 mL microtube and total EVs were sedimented via incubation with 126 µL of Exoquick™ (cat no. EXOQ100A-1; System Biosciences) for 60 min at RT, followed by centrifugation at 1500 × g for 30 min at RT. The crude EV pellet was resuspended in 350 µL of ultra-pure distilled water supplemented with protease/phosphatase inhibitors overnight with gentle rotation mixing at 4 °C. Crude EVs were incubated for 30 min at RT with 4 µg of biotinylated anti-human L1CAM antibody (clone 5G3) (cat. no. 13–1719-82; Thermo Fisher Scientific) or a three-antibody cocktail against canonical EV tetraspanins CD81 (cat. no. 302–030; Ancell), CD9 (cat. no. 558749; BD Pharmingen) and CD63 (cat. no. MAB15361; Abnova), to derive NDEVs or pan-tetraspanin expressing EVs (panTET-EVs), respectively. The EV-antibody complexes were then incubated with 25 µL of Pierce™ Streptavidin Plus UltraLink™ Resin (cat. no. 53117; Thermo Fisher Scientific) for 30 min at RT. After centrifugation at 600 × g for 10 min at 4 °C and removal of supernatant, NDEVs or panTET-EVs were eluted with 100 µL of 0.1 M glycine (stock solution at 1 M, pH = 2.7; cat. no. 24074–500; Polysciences, Inc.). Beads were sedimented by centrifugation at 4000 × g for 10 min at 4 °C, and supernatant containing immunoprecipitated EVs was transferred to a clean tube, where pH was immediately neutralized with 10 µL of 1 M tris hydrochloride (Tris–HCL, pH = 8; cat. no. CAS1185-53–1; Fisher Scientific). 10 µL of intact EVs were stored at − 80 °C for nanoparticle tracking analysis (NTA) and the remaining volume was subjected to EV lysis via two freeze–thaw cycles in 25 µL of 10% bovine serum albumin and 365 µL of 1X RIPA lysis buffer (stock solution at 10X; cat. no. 20–188; EMD Millipore Corp.) supplemented with 1X protease/phosphatase inhibitors. Lysed EVs were stored at − 80 °C.

### Biomarker determinations in NDEV lysates

We used enzyme-linked immunosorbent assays (ELISAs) to quantify the concentration of proBDNF (cat. no. BEK-2237; Biosensis) and humanin, also known as human putative humanin peptide or MT-RNR2 (cat. no. CSB-EL015084HU; Cusabio), in NDEV lysates; plates were read using the Synergy™ H1 microplate reader set to 450 nm and the Gen5™ microplate data collection software (BioTek Instruments). NDEV levels of BDNF, irisin, apelin, fractalkine, erythropoeitin (EPO), osteonectin (SPARC), interleukin-15 (IL-15), myostatin (MSTN)/GDF8, FABP3, follistatin-like 1 protein (FSTL-1), oncostatin-M (OSM) and osteocrin/musclin were quantified utilizing a Milliplex® Human Myokine Magnetic Bead panel (cat. no. HMYOMAG-56 K; EMD Millipore Corporation). Milliplex plates were read using the Luminex® 100/200™ instrument with the xPOTENT® acquisition software (Luminex Corporation). NDEV and panTET-EV samples were run undiluted based on experiments determining the optimal input volume for each assay. Standard curve equations of ELISAs were determined using four-parameter logistic (4-PL) regression, whereas for Milliplex assays, protein concentrations were extrapolated from a five-parameter logistic (5-PL) curve.

Samples were assessed in duplicate in all assays. The limit of detection (LOD) was defined as mean signal of the blank plus 2.5 times its standard deviation (SD). The lowest limit of quantification (LLOQ) was set by the following rules: (1) signal above LOD, (2) coefficient of variation (CV) > 20%, and (3) 80–120% recovery. A zero value was imputed to samples with signals below LOD; the LLOQ was imputed for signals between LOD and LLOQ; duplicate measurements with %CV ≥ 30 were excluded from the analysis. Excluded samples per analyte: 2 for proBDNF, 15 for humanin in NDEVs, 0 for humanin in panTET-EVs, 27 for apelin, 10 for fractalkine, 2 for BDNF, 2 for EPO, 8 for SPARC, 1 for IL-15, 3 for MSTN/GDF8, 13 for FABP3, 30 for irisin, 28 for FSTL-1, 21 for OSM and 21 for osteocrin/musclin.

An internal control (IC) was included in all plates to assess inter-plate variability. NDEVs from a healthy participant and a quality control provided were used as ICs for ELISAs and the Milliplex assay, respectively. CVs for the ICs across all plates were below 30% and hence, signals were not normalized (CVs for proBDNF, 9.9%; humanin, 23.1%; apelin, 5.3%; fractalkine, 11.8%; BDNF, 21.7%; EPO, 3.8%; SPARC, 8.4%; IL-15, 8.7%; MSTN/GDF8, 9.8%; FABP3, 12.0%; irisin, 3.6%; FSTL-1, 9.3%; OSM, 7.4%; and osteocrin/musclin, 17.3%).

## Results

Cohort demographics, clinical information, and cognitive performance measures at baseline are summarized in Table 1. Randomization successfully balanced groups for important parameters that could have acted as confounders of exercise effects.

**Table 1:** Cohort demographics

	*Control* (*n* = 47)	*Exercise* (*n* = 48)
*Age* (mean ± SD)	71.2 ± 6.5	71.3 ± 6.5
*Gender* (F/M)	20/28	19/28
*Body mass index* (mean ± SD)	23.9 ± 3.6	24.9 ± 4.0
*APOE status* (% ε4 positive)	67%	68%
*Baseline VO2max*	25.6 ± 8.5	25.3 ± 6.7
*Follow-up VO2max*	27.9 ± 9.5	29.4 ± 7.0
*Change from baseline VO2max*	0.5 ± 4.9	3.9 ± 5.6*
*Baseline MMSE* (mean ± SD)	24.4 ± 3.8	23.5 ± 3.3
*Baseline SDMT-120* (mean ± SD)	27.3 ± 14.6	25.7 ± 13.1
*Educational level* (%, more than 11 years of education)	44%	38%
*Smoking* (% currently smoking)	17%	15%
*Use of alcohol* (%, more than 2 units per day)	21%	11%
Anti-dementia medication (%)	94%	100%
Anti-depressant medication (%)	29%	26%
Anti-hypertensive medication (%)	35%	43%

^*^P = 0.011, *t*-test. For the rest of the comparisons, no significant differences were found between the control and exercise groups using *t*-test (continuous variables) or Pearson’s chi-squared test (categorical variables). *MMSE*, Mini-Mental State Examination. *SDMT-120*, correct responses in 120 s on Symbol Digit-Modalities Test. *VO2max*, the maximal oxygen uptake estimated by the submaximal Astrand test

### Exercise does not alter NDEV concentration and size

Previous findings have shown that plasma contains a high concentration of soluble L1CAM that could significantly interfere with the enrichment of NDEVs via L1CAM immunoprecipitation [26]. Hence, we first sought to validate the EV composition of our NDEV preparations by NTA and quantify the enrichment of L1CAM^+^ EVs via flow cytometry analysis (FCA), following established guidelines [27]. NTA showed that NDEVs had sizes between 50 and 450 nm, which is typical for a mixed population of exosomes and smaller microvesicles (Fig. S1C). NTA results were consistent with FCA of total EVs and NDEVs, with a violet side scatter (vSSC) signal range mainly within that of nanobeads under 500 nm (Fig. S1D). The achieved enrichment was determined by quantifying L1CAM^+^ nanoparticles before and after L1CAM immunoprecipitation using FCA (Fig. S1D–H). Among EVs gated based on the detection of blue succinimidyl ester (BSE) staining all EVs (Fig. S1D), ~ 0.5% were positive for L1CAM (Fig. S1E); this portion drastically increased to over 40% after L1CAM immunoprecipitation (Fig. S1F). A vast percentage of L1CAM^+^ EVs in NDEV preparations were double-positive for the canonical EV markers CD9, CD63, and CD81 (Fig. S1G), confirming their EV identity. The specificity of the anti-L1CAM antibody was confirmed by FCA of NDEVs labeled with an isotype control antibody showing the absence of EVs within the L1CAM gate (Fig. S1H). The abolition of detected events after treatment with NP40 detergent further demonstrated that FCA signals originated from membrane-enclosed nanoparticles (Fig. S1D).

Given variable reports regarding the acute effects of exercise on the concentration and size of total circulating EVs [28, 29], we examined the effect of 16-week exercise on NDEVs. There were no group differences or changes over time in the concentration (Fig. S1A) and particle size (Fig. S1B and S1C) of NDEVs based on NTA. To account for any effects of differential NDEV yield on NDEV protein biomarkers, the concentration of NDEVs was used as a covariate in all models, as previously done [30].

### Exercise increases proBDNF, BDNF, and humanin in NDEVs

**Fig. 1:**
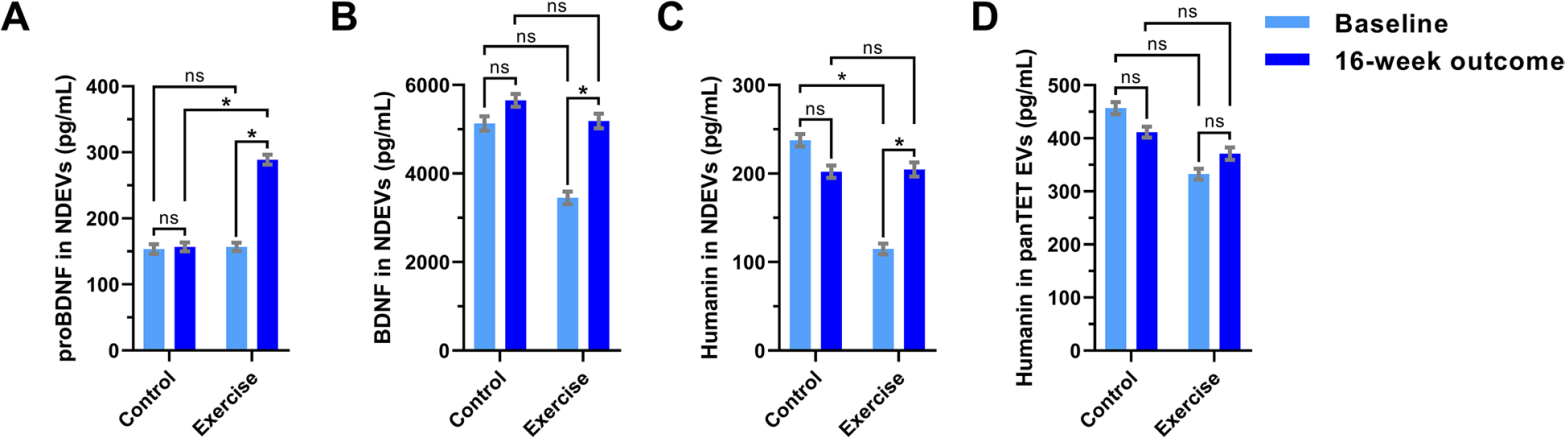
Neurotrophic and neuroprotective factors carried by NDEVs increase after exercise. Bar graphs show the concentrations of proBDNF (**A**), BDNF (**B**), and humanin (**C**) in NDEVs of control and exercise group participants at baseline (light blue) and 16 weeks (dark blue). **D** Humanin in EVs of all cellular origins immunocaptured by targeting panTET-EVs. Graphs show the estimated marginal mean ± standard error of the mean from repeated measures mixed-effects models. * indicates *p* < 0.05; ns: not significant

NDEV proBDNF, BDNF, and humanin increased in the exercise group after 16 weeks compared to baseline, whereas no differences over time were seen in the control group (Fig. 1A–C, Table 2). The greatest change occurred for proBDNF, which showed a 1.8-fold increase in the exercise group [from 139.7 (56.3–223 95% confidence interval; CI) to 274.7 (183.8–365.6 95% CI) pg/mL; *p* = 0.007] resulting higher in the exercise compared to the control group at 16 weeks [274.7 (183.8–365.6 95% CI) vs. 150.5 (65.5–235.5, 95% CI) pg/mL, *p* = 0.047]. NDEV humanin was lower at baseline in the exercise compared with the control group [91.88 (10.0–173.7 95% CI) vs. 232.8 (147.9–317.8 95% CI) pg/mL], but this difference disappeared after 16 weeks [184.0 (93.1–274.9 95% CI) vs. 194.1 (107.6–280.5 95% CI) pg/mL]. A similar trend at baseline was observed for BDNF. These observations suggest that for NDEV humanin and BDNF, randomization was not as efficient in eliminating baseline differences as it was for proBDNF, with very similar baseline levels between the control and exercise groups. Since humanin, unlike proBDNF/BDNF, is ubiquitously produced, we sought to also assess baseline differences and exercise-induced changes in humanin across all cells using panTET-EVs. PanTET-EV levels of humanin were no different at baseline and its levels remained unchanged within and between groups (Fig. 1D).

**Table 2:** Protein levels of proBDNF, BDNF, and humanin in NDEVs and panTET-EVs

*Analyte*	*Source*	*Randomization*	*Visit*	*Number of samples*	*Mean*^a^ *(pg/mL)*	*95% confidence interval*		Randomization*visit univariate test (within groups)				Randomization*visit univariate test (between groups)			
						*Lower bound*	*Upper bound*	*Numerator df*	*Denominator df*	*F*	*Significance*	*Numerator df*	*Denominator df*	*F*	*Significance*
proBDNF	NDEVs	Control	Baseline	38	148.79	60.81	236.76								
			16-week outcome	43	150.49	65.49	235.48	1	77.255	0.001	0.973				
		Exercise	Baseline	44	139.679	56.29	223.06					1	130.961	0.022	0.882
			16-week outcome	37	274.7	183.84	365.55	1	76.073	7.802	**0.007**	1	133.047	4.03	**0.047**
BDNF	NDEVs	Control	Baseline	37	5091.51	3159.8	7023.22								
			16-week outcome	43	5523.43	3631.36	7415.5	1	53.51	0.31	0.58				
		Exercise	Baseline	43	3201.27	1319.87	5082.68					1	104.878	1.962	0.164
			16-week outcome	36	4918.05	2926.75	6909.35	1	52.512	5.033	**0.029**	1	107.904	0.198	0.657
Humanin	NDEVs	Control	Baseline	38	232.83	147.9	317.77								
			16-week outcome	40	194.07	107.6	280.54	1	53.549	1.023	0.316				
		Exercise	Baseline	44	91.88	10.04	173.72					1	102.06	5.626	**0.02**
			16-week outcome	33	183.97	93.05	274.88	1	53.267	5.933	**0.018**	1	113.739	0.026	0.872
Humanin	panTET-EVs	Control	Baseline	38	465.86	316.19	615.52								
			16-week outcome	43	412.17	265.42	558.91	1	77.183	0.477	0.492				
		Exercise	Baseline	44	313.12	169.76	456.48					1	127.573	2.139	0.146
			16-week outcome	37	356.17	199.92	512.42	1	75.853	0.32	0.573	1	131.16	0.277	0.6

^a^ Estimated marginal means adjusted by age, sex, EV concentration, and APOE status

Correlations with *P* values in bold are statistically significant

**Fig. 2:**
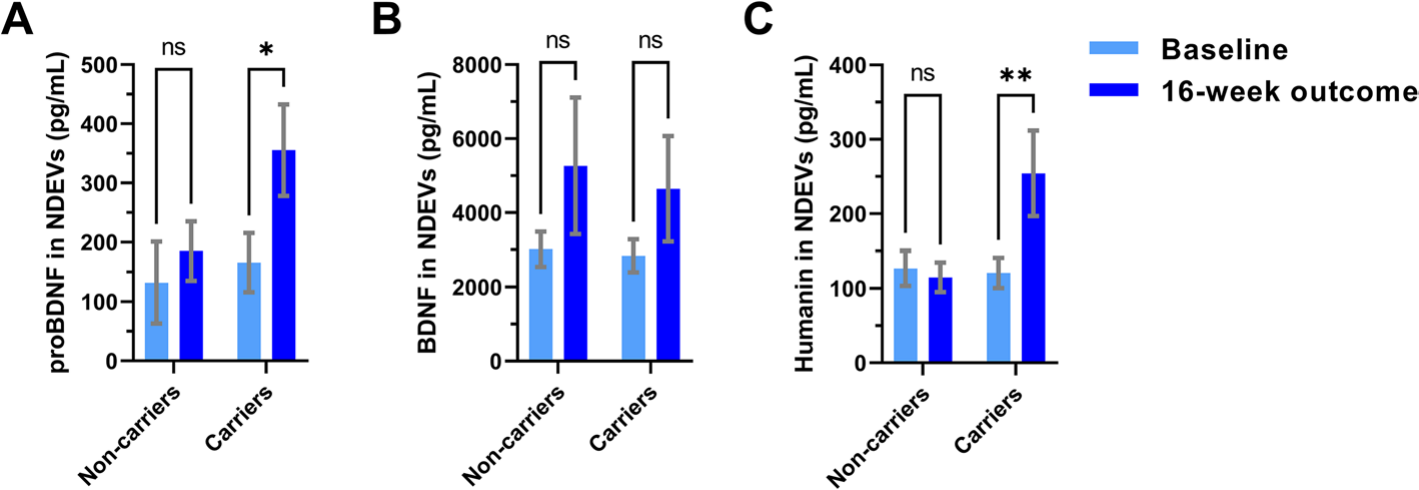
NDEV exercise effects stratified by APOE ε4 genotype. Bar graphs show the concentrations of proBDNF (**A**), BDNF (**B**), and humanin (**C**) in NDEVs from ε4 carriers and non-carriers from the exercise group at baseline (light blue) and 16 weeks (dark blue). Graphs show the estimated marginal mean ± standard error of the mean from repeated measures mixed-effects models. * indicates *p* < 0.05; ns: not significant

In an exploratory analysis, we examined exercise effects in stratified sub-groups of apolipoprotein-E (APOE) ε4 carriers and non-carriers (Table S1), as has recently been suggested by multiple experts in the AD field [31]. Interestingly, in the exercise group, NDEV levels of proBDNF and humanin increased only in ε4 carriers; proBDNF: ε4 non-carriers [from 131.9 (− 17.1–281.0 95% CI) to 185.2 (77.0–293.5 95% CI) pg/mL; *p* = 0.577], ε4 carriers [from 165.9 (63.7–268.1 95% CI) to 355.5 (194.9–516.0 95% CI) pg/mL; *p* = 0.016]; humanin: ε4 non-carriers [from 127.0 (76.4–177.5 95% CI) to 115.0 (70.5–159.6 95% CI) pg/mL; *p* = 0.858], ε4 carriers [from 120.8 (79.0–162.5 95% CI) to 254.3 (134.1–374.4 95% CI) pg/mL; *p* = 0.005 (Fig. 2A and C). No significant changes were observed for BDNF in this stratified analysis (Fig. 2B).

### NDEV biomarkers of exercise are inter-related

As physical activity can profoundly alter the neuronal proteome [32], we explored whether exercise modulates NDEV cargo for exerkines expressed by neurons (as confirmed by https://www.proteinatlas.org/) (Table S2). We were particularly interested in irisin, an exercise-induced hormone shown to regulate cognitive function by promoting BDNF expression [33]. Although no changes were observed in NDEV irisin with exercise, as was also the case for all exerkines (Table S2), we observed a positive correlation with BDNF at baseline that was preserved at 16 weeks for both groups (Fig. S2C; Tables S3 and S4). Similar positive correlations were observed between levels of NDEV irisin and humanin and between humanin and proBDNF (Fig. S2A and B; Tables S3 and S4). We also examined the relationships between exercise-induced changes in NDEV biomarkers and the only significant association observed was between the change in NDEV irisin and that of BDNF in the exercise group (Fig. S2D; Table S5).

## Discussion

In this study, we sought to shed light on the molecular mechanisms underlying the beneficial effects of exercise in AD by examining a priori hypothesized molecular effectors in plasma NDEVs from AD patients participating in a RCT of exercise. We found that in patients with mild to moderate AD, 16 weeks of aerobic exercise increased NDEV levels of BDNF, proBDNF, and humanin (Fig. 1). These findings strengthen the notion that NDEV cargo reflects the molecular state of brain neurons and any dynamic changes to it, a thesis supported by previous evidence showing that NDEV biomarkers can track AD progression [34] and demonstrate target engagement in clinical trials [20].

BDNF is a neurotrophin produced upon proteolytic cleavage of its precursor, proBDNF, which is also bioactive. Physical activity increases BDNF concentrations in brain [35] and plasma [36]. Previous studies have shown that proBDNF and mature BDNF mRNA and protein, are decreased in early and end-stage AD brain in correlation with cognitive measures [14]. The upregulation of BDNF by exercise has been observed in both animal [37] and human studies [38] supporting its development as a non-pharmacological intervention for AD. Proposed mechanisms on how exercise may increase BDNF levels in neurons include: (i) effects through the PGC1α-dependent myokine, irisin [33], (ii) the myokine Cathepsin B, which when increased peripherally by exercise can cross the BBB and enhance BDNF production and hence neurogenesis [39] and (iii) β-hydroxybutyrate, which also increases during aerobic exercise and increases BDNF expression in the brain [37]. As previously shown, both proBDNF and BDNF are present in NDEVs, at higher levels compared to plasma [40]. Moreover, NDEV proBDNF, but not BDNF, was associated with physical activity in a large longitudinal cohort of aging [41]. Future studies, unrestrained by the amount of plasma available, may use NDEVs as a tool to further dissect the downstream effects of proBDNF and BDNF, such as by measuring their receptor levels (p75 NTR and TrkB), downstream effectors and functional outcomes, such as levels of synaptic proteins [34].

Humanin is a mitochondria-derived peptide that suppresses neuronal apoptosis, preserves synapses, reduces inflammation, and supports glucose and oxidative metabolism [42]. Plasma humanin decreases with age in humans and mice [43], and upon replenishment, cognition in aged mice is improved [44]. Humanin mRNA in plasma EVs has been found decreased in AD compared to control individuals [45], whereas protein levels of humanin in NDEVs have been found decreased in multiple neuropsychiatric disorders [46, 47]. Interestingly, physical activity increases humanin in plasma [48]. Humanin can be destabilized via ubiquitination assisted by TRIM11 [49], a process that could be interrupted by aerobic exercise, which inhibits the ubiquitin-proteosome pathway [50]. In turn, increased humanin levels could inhibit the pro-apoptotic factors BAX and BID, thus preventing mitochondrial-outer membrane permeabilization and enhancing neuronal survival and cognitive performance in AD [51]. These observations suggesting that humanin depletion is associated with mitochondrial function deficits in AD further support humanin augmentation via physical activity as a non-pharmacological intervention for AD.

Exercise-induced factors released from muscle and other organs, collectively coined exerkines, can mediate systemic responses to physical activity [13]. The extracellular milieu is a harsh environment for soluble exerkines, which may have driven the evolution of a parallel lipid-enclosed delivery mode via EVs, which are known to exert autocrine, paracrine, and endocrine signaling functions [13]. Previous studies have shown that endurance exercise increases circulating EVs, including a muscle-derived subpopulation capable of being biodistributed to the brain after peripheral injection [13, 52]. Thus, it has been widely hypothesized that physical activity modulates neuronal function indirectly, through the activity of soluble or EV-associated peripheral exerkines capable of crossing the BBB. Although many of these peripheral exerkines are also expressed in neurons, they remained unchanged in NDEVs after exercise (Table S2), suggesting that physical activity engages different pathways in neurons and peripheral organs.

Our study also indicates that neuronal exercise effects are coordinated across multiple inter-connected pathways; this is suggested by the correlation between changes in NDEV BDNF and irisin (Fig. S2), proteins whose expression is known to be co-regulated [53], as well as correlations between humanin with irisin and proBDNF, both at baseline and after exercise, (Fig. S2) which have not been previously reported.

We explored whether exercise effects may differ by APOE genotype, as previous studies have shown that, in both healthy elderly and AD individuals, beneficial effects of physical activity on cognition and hippocampal volume are stronger in ε4 carriers [54]. These studies suggest that the presence of ε4 increases the responsiveness of neurons to exercise. In favor of such a hypothesis, we found more prominent increases in NDEV proBDNF and humanin with exercise in ε4 carriers (Fig. 2).

## Limitations

The randomized controlled design of ADEX offers the highest level of clinical evidence, especially given a relatively large *N*, rigorous inclusion criteria including PET-PiB brain imaging and good adherence [4]. Sixty-two percent of the patients adhered to the exercise intervention both in terms of attendance (attended more than 80% of planned visits) and exercise intensity (exercised at more than 70% of maximal heart rate during sessions) [4]. Nevertheless, intrinsic generalizability constraints limit the relevance of our results to patients with mild to moderate AD. Also, the present study was limited in terms of enrolling a racially and ethnically homogeneous Danish population; future studies should aim to increase cohort diversity to assess the potential effects of racial and ethnic backgrounds and relevant disparities. Moreover, results may not be extrapolated to interventions of different exercise duration, intensity, and type. Our study was also constrained by the amount of available plasma, which limited the number of biomarkers we were able to quantify. A further limitation is that most of the participants in this study had a normal body mass index (BMI) (Table 1). Therefore, it would be very interesting to assess the effects of exercise in NDEV biomarkers in overweight and obese individuals. Increased BMI is associated with neuroinflammation [55] and increased risk of dementia in older age [56]. Our results motivate the hypothesis that both the beneficial cognitive and neuropsychological effects of physical activity and the corresponding increase in NDEV proBDNF, BDNF, and humanin may be enhanced in individuals with AD and increased BMI. This hypothesis should be addressed in future studies leveraging study cohorts that enroll individuals with high BMI and/or related comorbidities, such as insulin resistance or metabolic syndrome.

## Conclusions

The present study offers a mechanistic basis for the beneficial effects of aerobic exercise in early AD by implicating the upregulation of proBDNF/BDNF and humanin. To further advance our understanding of the effects of non-pharmacological treatments for AD and related dementias, future studies should leverage samples from RCTs of exercise alone and/or multidomain lifestyle interventions and examine the relationships of EV biomarkers with cognitive and functional outcomes.

### Supplementary Information

**Additional file 1:** **Supplementary Figure 1.** Characterization of NDEVs. A and B) Box plots show the concentration (A) in particles/mL and size mode (B) in nanometers (nm) of NDEVs from the plasma of subjects in the control and exercise groups at baseline (light blue) and at the 16-week outcome (dark blue) determined using nanoparticle tracking analysis (NTA). Control group: baseline, N=37; 16-week outcome, N=37. Exercise group: baseline, N=42; 16-week outcome, N=34. Statistical analyses: mixed-effects linear model with Fisher’s LSD test. C) Line graphs of the particle percentage in function of particle size (nm) determined using NTA show representative size profiles of NDEVs from subjects in the exercise group at baseline (light blue; N=10) and at the 16-week outcome (dark blue; N=10) visits. D to H) High-sensitivity nanoscale multiplex flow cytometry analysis (FCA) of pooled crude plasma EVs from multiple subjects sedimented using ExoQuick® before and after immunoprecipitation of NDEVs. Dot plots show the violet size scatter (vSSC) in function of the fluorescent signal of samples co-labeled with the fluorescent EV marker blue succinimidyl ester (BSE) (violet events in D) and PE-tagged anti-L1CAM antibody (E and F). In D, a gate designated based on the background signal of negative controls encloses events positive for BSE in crude EVs, with similar results obtained for NDEVs (data not shown). The vSSC vs. BSE signal sensitivity to treatment with NP40 detergent (yellow events in D) confirms the membranous composition of detected events. A color-coded size range based on the vSSC of FITC-tagged beads is included on the right for the size comparison of events. Plots E and F compare the percentage of L1CAM-positive events in crude EVs and NDEVs, respectively, determined by the abundance of BSE-gated events within L1CAM-PE gates established based on the background signal of a negative control not incubated with anti-L1CAM antibody (yellow events in E). In G, a dot plot shows BSE-gated events of NDEVs co-stained with antibodies against L1CAM (PE-tagged, x-axis) and pan-Tetraspanins CD9, CD63 and CD81 (APC-tagged, y-axis). A histogram in H shows the abundance of BSE-gated PE-positive events in NDEVs labelled with PE-tagged anti-L1CAM antibody (turquoise) or its isotype control (yellow). The frequency of events in analyzed samples did not result in coinciding events as confirmed by swarming experiments (data not shown). **Supplementary table 1.** NDEV protein levels in the exercise group by APOE4 status. **Supplementary Figure 2.** NDEV protein level correlations are maintained after exercise. A to C) Scatter plots show the association between NDEV levels of selected protein pairs with significant correlations both at baseline (left) and at the 16-week outcome (right): (A) humanin in function of proBDNF; (B) Humanin in function of Irisin; (C) BDNF in function of Irisin. In D, a scatter plot shows the relationship between BDNF and irisin protein level changes from baseline to the 16-week outcome for the control (light blue) and exercise groups (dark blue). Plots show the ‘r’ and P values of nonparametric Spearman correlations. **Supplementary table 2.** Protein levels of putative exerkines in NDEV lysates. **Supplementary table 3.** Correlations between analyte levels in NDEVs at baseline. **Supplementary table 4.** Correlations between analyte levels in NDEVs at 16-week outcome. **Supplementary table 5.** Correlations between analyte level changes in NDEVs from baseline to 16-week outcome.

## Data Availability

The datasets used and/or analyzed during the current study are available from the corresponding author on reasonable request.

## Abbreviations

4-PL: Four-parametric logistic
5-PL: Five-parametric logistic
AD: Alzheimer’s disease
ADEX: Effect of Physical Exercise in Alzheimer Patients (brief) or Preserving Cognition, Quality of Life, Physical Health and Functional Ability in Alzheimer's Disease: The Effect of Physical Exercise
APOE: Apolipoprotein-E
BBB: Blood-brain barrier
BDNF: Brain-derived neurotrophic factor
BMI: Body mass index
BSE: Blue succinimidyl ester
CI: Confidence interval
CSF: Cerebrospinal fluid
CV: Coefficient of variation
DPBS: Dulbecco’s phosphate-buffered saline
ELISA: Enzyme-linked immunosorbent assay
EPO: Erythropoeitin
EV: Extracellular vesicles
FCA: Flow-cytometry analysis
FSTL-1: Follistatin-like 1 protein
IC: Internal control
IL-15: Interleukin-15
L1CAM: L1 cell adhesion molecule
LLOQ: Lowest limit of quantification
LOD: Limit of detection
MSTN: Myostatin
NDEV: Neuron-derived extracellular vesicles
NfL: Light chain neurofilaments
NTA: Nanoparticle tracking analysis
OSM: Oncostatin-M
panTET-EV: Pan-tetraspanin extracellular vesicles
RCT: Randomized controlled trial
RT: Room temperature
SD: Standard deviation
SDMT: Symbol digit modalities test
SPARC: Osteonectin
Tris-HCL: Tris hydrochloride
vSSC: Violet side scatter
