# Correction: Neuron‑derived extracellular vesicles in blood reveal effects of exercise in Alzheimer’s disease

**DOI:** 10.1186/s13195-023-01371-x

**Published:** 2024-01-23

**Authors:** Francheska Delgado‑Peraza, Carlos Nogueras‑Ortiz, Anja Hviid Simonsen, De’Larrian DeAnté Knight, Pamela J. Yao, Edward J. Goetzl, Camilla Steen Jensen, Peter Høgh, Hanne Gottrup, Karsten Vestergaard, Steen Gregers Hasselbalch, Dimitrios Kapogiannis

**Affiliations:** 1 Laboratory of Clinical Investigation, Intramural Research Program, National Institute On Aging, National Institutes of Health, Baltimore, MD 21224 USA; 2 Danish Dementia Research Centre, Copenhagen University Hospital - Rigshospitalet, 2100 Copenhagen, Denmark; 3 Department of Medicine, University of California San Francisco, San Francisco, CA 94143 USA; 4 Research Department, Campus for Jewish Living, San Francisco, CA 94112 USA; 5 Department of Neurology, Zealand University Hospital, 4000 Roskilde, Denmark; 6 Department of Clinical Medicine, University of Copenhagen, 1165 Copenhagen, Denmark; 7 Department of Neurology, Dementia Clinic, Aarhus University Hospital, 8200 Aarhus, Denmark; 8 Department of Neurology, Dementia Clinic, Aalborg University Hospital, 9000 Aalborg, Denmark

**CorrectionAlz Res Therapy 15, 156 (2023)**

**https://doi.org/10.1186/s13195-023-01303-9**

Following the publication of the original article [1], the author reported that “Additional file 1” file in the published article is not the correct file.

The original article [1] has been updated.
