# Supplementary material for: Neuron-derived extracellular vesicles in blood reveal effects of exercise in Alzheimer’s disease
Source: Alzheimers Res Ther. 2023 Sep 20;15:156. doi: 10.1186/s13195-023-01303-9 (PMC10510190; doi:10.1186/s13195-023-01303-9)
Supplement: Supplementary file 1 — Additional file 1: Supplementary Figure 1. Characterization of NDEVs. A and B) Box plots show the concentration (A) in particles/mL and size mode (B) in nanometers (nm) of NDEVs from the plasma of subjects in the control and exercise groups at baseline (light blue) and at the 16-week outcome (dark blue) determined using nanoparticle tracking analysis (NTA). Control group: baseline, N=37; 16-week outcome, N=37. Exercise group: baseline, N=42; 16-week outcome, N=34. Statistical analyses: mixed-effects linear model with Fisher’s LSD test. C) Line graphs of the particle percentage in function of particle size (nm) determined using NTA show representative size profiles of NDEVs from subjects in the exercise group at baseline (light blue; N=10) and at the 16-week outcome (dark blue; N=10) visits. D to H) High-sensitivity nanoscale multiplex flow cytometry analysis (FCA) of pooled crude plasma EVs from multiple subjects sedimented using ExoQuick® before and after immunoprecipitation of NDEVs. Dot plots show the violet size scatter (vSSC) in function of the fluorescent signal of samples co-labeled with the fluorescent EV marker blue succinimidyl ester (BSE) (violet events in D) and PE-tagged anti-L1CAM antibody (E and F). In D, a gate designated based on the background signal of negative controls encloses events positive for BSE in crude EVs, with similar results obtained for NDEVs (data not shown). The vSSC vs. BSE signal sensitivity to treatment with NP40 detergent (yellow events in D) confirms the membranous composition of detected events. A color-coded size range based on the vSSC of FITC-tagged beads is included on the right for the size comparison of events. Plots E and F compare the percentage of L1CAM-positive events in crude EVs and NDEVs, respectively, determined by the abundance of BSE-gated events within L1CAM-PE gates established based on the background signal of a negative control not incubated with anti-L1CAM antibody (yellow events in E). In G, a dot plot sh [file 13195_2023_1303_MOESM1_ESM.pdf]

**Supplementary Figures and Tables**: *Delgado-Peraza F, Nogueras-Ortiz C, Simonsen AH, Knight DD, Yao PJ, Goetzl EJ, Jensen CS, Høgh P, Gottrup H, Vestergaard K, Hasselbalch SG, Kapogiannis D. Neuron-derived extracellular vesicles in blood reveal effects of exercise in Alzheimer's disease. Alzheimers Res Ther. 2023 Sep 20;15(1):156. doi: 10.1186/s13195-023-01303-9. PMID: 37730689; PMCID: PMC10510190.*

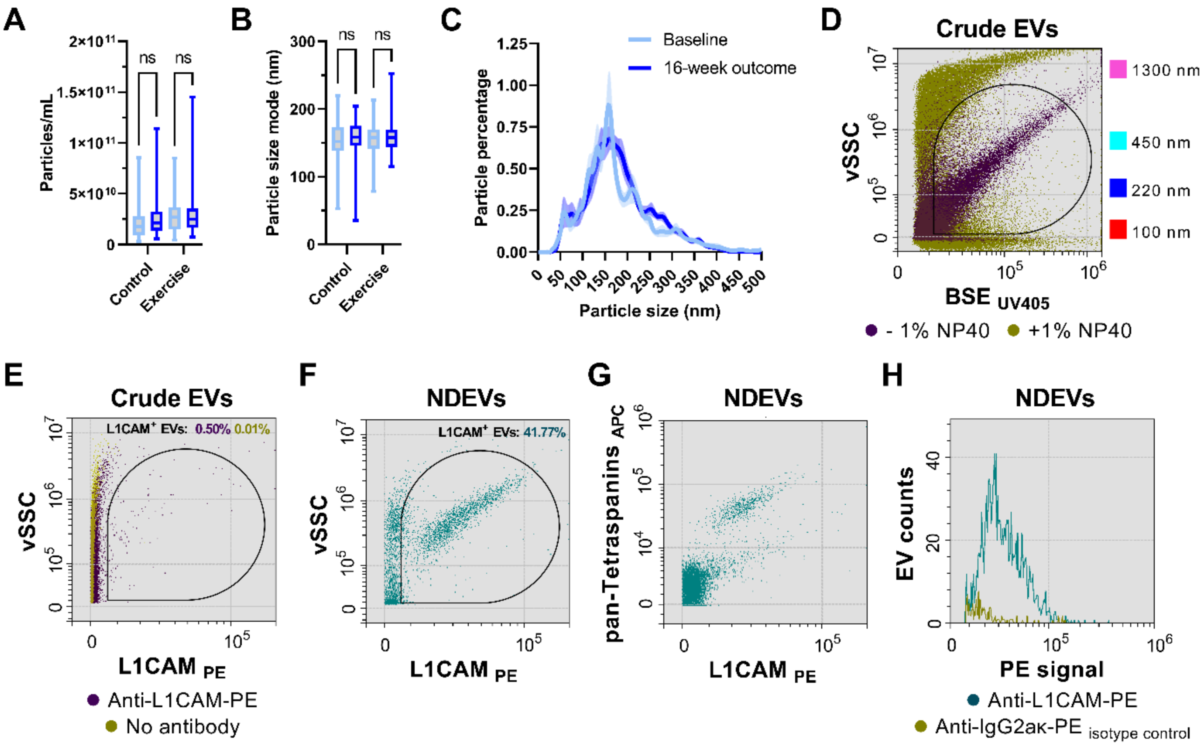


**Supplementary Figure 1. Characterization of NDEVs. A and B)** Box plots show the concentration (A) in particles/mL and size mode (B) in nanometers (nm) of NDEVs from the plasma of subjects in the control and exercise groups at baseline (light blue) and at the 16-week outcome (dark blue) determined using nanoparticle tracking analysis (NTA). Control group: baseline, N=37; 16-week outcome, N=37. Exercise group: baseline, N=42; 16-week outcome, N=34. Statistical analyses: mixed-effects linear model with Fisher's LSD test. **C)** Line graphs of the particle percentage in function of particle size (nm) determined using NTA show representative size profiles of NDEVs from subjects in the exercise group at baseline (light blue; N=10) and at the 16-week outcome (dark blue; N=10) visits. **D to H)** High-sensitivity nanoscale multiplex flow cytometry analysis (FCA) of pooled crude plasma EVs from multiple subjects sedimented using ExoQuick® before and after immunoprecipitation of NDEVs. Dot plots show the violet size scatter (vSSC) in function of the fluorescent signal of samples co-labeled with the fluorescent EV marker blue succinimidyl ester (BSE) (violet events in **D**) and PE-tagged anti-

L1CAM antibody (**E** and **F**). In **D**, a gate designated based on the background signal of negative controls encloses events positive for BSE in crude EVs, with similar results obtained for NDEVs (data not shown). The vSSC vs. BSE signal sensitivity to treatment with NP40 detergent (yellow events in **D**) confirms the membranous composition of detected events. A color-coded size range based on the vSSC of FITC-tagged beads is included on the right for the size comparison of events. Plots **E** and **F** compare the percentage of L1CAM-positive events in crude EVs and NDEVs, respectively, determined by the abundance of BSE-gated events within L1CAM-PE gates established based on the background signal of a negative control not incubated with anti-L1CAM antibody (yellow events in **E**). In **G**, a dot plot shows BSE-gated events of NDEVs co-stained with antibodies against L1CAM (PE-tagged, x-axis) and pan-Tetraspanins CD9, CD63 and CD81 (APC-tagged, y-axis). A histogram in **H** shows the abundance of BSE-gated PE-positive events in NDEVs labelled with PE-tagged anti-L1CAM antibody (turquoise) or its isotype control (yellow). The frequency of events in analyzed samples did not result in coinciding events as confirmed by swarming experiments (data not shown).

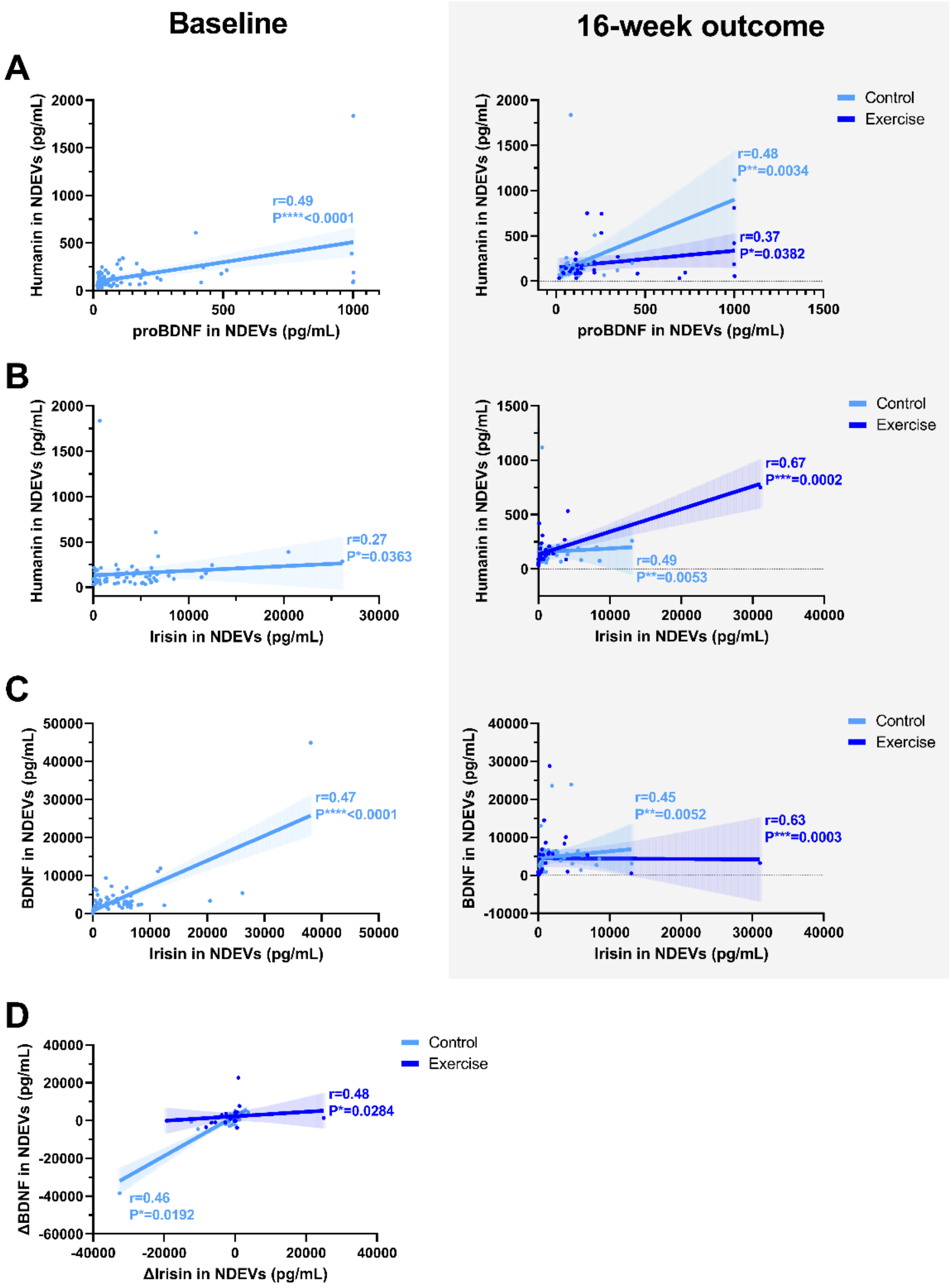


**Supplementary Figure 2. NDEV protein level correlations are maintained after exercise. A to C)** Scatter plots show the association between NDEV levels of selected protein pairs with significant correlations both at baseline (left) and at the 16-week outcome (right): (**A**) Humanin in function of proBDNF; (**B**) Humanin in function of Irisin; (**C**) BDNF in function of Irisin. In **D**, a scatter plot shows the relationship between BDNF and Irisin protein level changes from baseline

to the 16-week outcome for the control (light blue) and exercise groups (dark blue). Plots show the ‘r’ and P values of nonparametric Spearman correlations.

**Supplementary table 1.** NDEV protein levels in the exercise group by APOE4 status

| *Analyte* | *APOE4 status* | *Visit* | *Number of samples* | *Mean[†] (pg/mL)* | *95% Confidence Interval* | |
|---|---|---|---|---|---|---|
| | | | | | *Upper Bound* | *Lower Bound* |
| proBDNF | Non-carriers | Baseline | 14 | 131.93 | 280.95 | -17.09 |
| | | 16-week outcome | 14 | 185.23 | 293.48 | 76.98 |
| | Carriers | Baseline | 29 | 165.89 | 268.11 | 63.67 |
| | | 16-week outcome | 23 | 355.45 | 516.04 | 194.86 |
| BDNF | Non-carriers | Baseline | 15 | 3018.18 | 4036.75 | 1999.60 |
| | | 16-week outcome | 14 | 5265.43 | 9245.15 | 1285.71 |
| | Carriers | Baseline | 28 | 2842.15 | 3751.02 | 1933.27 |
| | | 16-week outcome | 22 | 4645.19 | 7603.65 | 1686.74 |
| Humanin | Non-carriers | Baseline | 14 | 126.98 | 177.52 | 76.43 |
| | | 16-week outcome | 11 | 115.00 | 159.55 | 70.45 |
| | Carriers | Baseline | 29 | 120.77 | 162.54 | 79.01 |
| | | 16-week outcome | 20 | 254.25 | 374.44 | 134.07 |

[†] Estimated marginal means adjusted by age, sex, EV concentration and APOE status

**Supplementary table 2.** Protein levels of putative exerkines in NDEV lysates

| Analyte | Neuronal RNA expression (TPM)† | Randomization | Visit | Number of samples | Mean†† (pg/mL or **ng/mL**) | 95% Confidence Interval | | Randomization*Visit Fixed Effects Test | | | |
|---|---|---|---|---|---|---|---|---|---|---|---|
| | | | | | | Lower Bound | Upper Bound | Numerator df | Denominator df | F | Significance |
| Fractalkine | 24.3 ± 3.8 | Control | Baseline | 35 | 127.6 | 81.98 | 173.23 | 1 | 63.9 | 0.655 | 0.42 |
| | | | 16-week outcome | 40 | 87.55 | 43.69 | 131.41 | | | | |
| | | Exercise | Baseline | 42 | 65.07 | 21.81 | 108.32 | | | | |
| | | | 16-week outcome | 33 | 50.75 | 3.38 | 98.12 | | | | |
| Fatty acid binding protein 3 (FABP3) | 15.4 ± 0.8 | Control | Baseline | 33 | 400.88 | 218.47 | 583.3 | 1 | 77.95 | 0.281 | 0.6 |
| | | | 16-week outcome | 41 | 362.3 | 194.55 | 530.04 | | | | |
| | | Exercise | Baseline | 40 | 340.74 | 177.04 | 504.44 | | | | |
| | | | 16-week outcome | 34 | 397.93 | 214.95 | 580.9 | | | | |
| FNDC5/Irisin | 13.1 ± 2.8 | Control | Baseline | 29 | 4831.18 | 2644.17 | 7018.17 | 1 | 61.8 | 0.294 | 0.59 |
| | | | 16-week outcome | 37 | 2373.75 | 745.57 | 4601.92 | | | | |
| | | Exercise | Baseline | 35 | 4279.87 | 2294.31 | 6265.43 | | | | |
| | | | 16-week outcome | 30 | 3100.52 | 901.01 | 5300.03 | | | | |
| Follistatin-like 1 protein (FSTL-1) | 12.9 ± 3.3 | Control | Baseline | 32 | 202763.42 | 96341.15 | 309185.68 | 1 | 40.26 | 1.08 | 0.31 |
| | | | 16-week outcome | 36 | 133786.86 | 30145.4 | 237428.32 | | | | |
| | | Exercise | Baseline | 33 | 110765.42 | 2565.36 | 218965.48 | | | | |
| | | | 16-week outcome | 32 | 102640.18 | -7811.25 | 213091.62 | | | | |
| Osteonectin | 3.6 ± 0.7 | Control | Baseline | 37 | **8.79** | -49.87 | 67.44 | 1 | 94.58 | 1.57 | 0.21 |
| | | | 16-week outcome | 39 | **4.82** | -54.5 | 64.14 | | | | |
| | | Exercise | Baseline | 43 | **-2.28** | -57.75 | 53.2 | | | | |
| | | | 16-week outcome | 34 | **67.83** | 4.69 | 130.97 | | | | |
| Osteocrin | 3.5 ± 2.5 | Control | Baseline | 27 | 232.01 | 139.18 | 324.84 | 1 | 33.41 | 0.11 | 0.74 |
| | | | 16-week outcome | 34 | 168.44 | 81.31 | 255.57 | | | | |
| | | Exercise | Baseline | 28 | 112.4 | 23.14 | 201.66 | | | | |
| | | | 16-week outcome | 30 | 64.79 | -25.73 | 155.32 | | | | |
| Interleukin-15 | 3.4 ± 1.4 | Control | Baseline | 37 | 1.93 | 0.03 | 3.84 | 1 | 68.61 | 0.1 | 0.76 |
| | | | 16-week outcome | 43 | 1.26 | -0.59 | 3.1 | | | | |
| | | Exercise | Baseline | 44 | 1.6 | -0.16 | 3.36 | | | | |
| | | | 16-week outcome | 36 | 0.31 | -1.68 | 2.29 | | | | |
| Myostatin | 1.3 ± 0.1 | Control | Baseline | 37 | 939.52 | 184.36 | 1694.67 | 1 | 94.78 | 1.27 | 0.26 |
| | | | 16-week outcome | 42 | 111.91 | -621.56 | 845.38 | | | | |

| | | | | | | | | | | | |
|---|---|---|---|---|---|---|---|---|---|---|---|
| | | Exercise | Baseline | 43 | -36.37 | -736.42 | 663.69 | | | | |
| | | | 16-week outcome | 36 | -25.38 | -813.27 | 762.52 | | | | |
| Apelin | 0.3 ± 0.2 | Control | Baseline | 31 | 7463.84 | 1505.38 | 13422.3 | 1 | 36.99 | 0.05 | 0.83 |
| | | | 16-week outcome | 38 | 4865.52 | -650.66 | 10381.69 | | | | |
| | | Exercise | Baseline | 35 | 5766.9 | 132.88 | 11400.91 | | | | |
| | | | 16-week outcome | 30 | 2183.91 | -4001.79 | 8369.6 | | | | |
| Oncostatin M | 0.1 ± 0.0 | Control | Baseline | 32 | 44.22 | 13.29 | 75.14 | 1 | 89.53 | 0.29 | 0.59 |
| | | | 16-week outcome | 39 | 15.97 | -12.35 | 44.28 | | | | |
| | | Exercise | Baseline | 37 | 22.07 | -6.79 | 50.93 | | | | |
| | | | 16-week outcome | 32 | 9.55 | -22.17 | 41.27 | | | | |
| Erythropoeitin | 0.1 ± 0.0 | Control | Baseline | 22 | 862.12 | 482.82 | 1241.42 | 1 | 43.39 | 0.21 | 0.65 |
| | | | 16-week outcome | 28 | 639.96 | 282.48 | 997.43 | | | | |
| | | Exercise | Baseline | 23 | 881.76 | 493.26 | 1270.27 | | | | |
| | | | 16-week outcome | 16 | 826.57 | 374.42 | 1278.72 | | | | |

† Nucleotide transcripts per million (TPM) data extracted from The Human Protein Atlas

†† Estimated marginal means adjusted by age, sex, EV concentration and APOE status

**Supplementary table 3.** Correlations between analyte levels in NDEVs at baseline

| | *Analyte* | | | | | | | | | | | |
|---|---|---|---|---|---|---|---|---|---|---|---|---|
| | *proBDNF* | | | | *BDNF* | | | | *Humanin* | | | |
| *Analyte* | *Spearman r* | *95% C.I.†* | *P value* | *Sample size* | *Spearman r* | *95% C.I.†* | *P value* | *Sample size* | *Spearman r* | *95% C.I.†* | *P value* | *Sample size* |
| BDNF | 0.17 | -0.07 to 0.38 | 0.14876 | 78 | - | - | - | - | - | - | - | - |
| Humanin | 0.49 | 0.29 to 0.64 | **0.00001** | 77 | 0.17 | -0.07 to 0.39 | 0.14665 | 77 | - | - | - | - |
| Irisin | 0.16 | -0.11 to 0.40 | 0.22948 | 61 | 0.47 | 0.26 to 0.66 | **0.00009** | 63 | 0.27 | 0.02 to 0.50 | **0.03628** | 61 |

† Confidence interval

- redundant

Correlations with P values in bold are statistically significant

**Supplementary table 4.** Correlations between analyte levels in NDEVs at 16-week outcome

***Control group***

| | *Analyte* | | | | | | | | | | | |
|---|---|---|---|---|---|---|---|---|---|---|---|---|
| | *proBDNF* | | | | *BDNF* | | | | *Humanin* | | | |
| *Analyte* | *Spearman r* | *95% C.I.*† | *P value* | *Sample size* | *Spearman r* | *95% C.I.*† | *P value* | *Sample size* | *Spearman r* | *95% C.I.*† | *P value* | *Sample size* |
| BDNF | 0.21 | -0.10 to 0.49 | 0.16701 | 43 | - | - | - | - | - | - | - | - |
| Humanin | 0.48 | 0.16 to 0.70 | **0.00336** | 36 | 0.29 | -0.06 to 0.57 | 0.08379 | 36 | - | - | - | - |
| Irisin | 0.21 | -0.13 to 0.51 | 0.21048 | 37 | 0.45 | 0.14 to 0.68 | **0.00518** | 37 | 0.49 | 0.15 to 0.72 | **0.00533** | 31 |

***Exercise group***

| | *Analyte* | | | | | | | | | | | |
|---|---|---|---|---|---|---|---|---|---|---|---|---|
| | *proBDNF* | | | | *BDNF* | | | | *Humanin* | | | |
| *Analyte* | *Spearman r* | *95% C.I.*† | *P value* | *Sample size* | *Spearman r* | *95% C.I.*† | *P value* | *Sample size* | *Spearman r* | *95% C.I.*† | *P value* | *Sample size* |
| BDNF | -0.11 | -0.43 to 0.24 | 0.53556 | 36 | - | - | - | - | - | - | - | - |
| Humanin | 0.37 | 0.01 to 0.65 | **0.03823** | 31 | 0.32 | -0.06 to 0.61 | 0.08368 | 31 | - | - | - | - |
| Irisin | 0.00 | -0.38 to 0.38 | 0.98580 | 29 | 0.63 | 0.33 to 0.81 | **0.00025** | 29 | 0.67 | 0.38 to 0.85 | **0.00017** | 26 |

† Confidence interval

- redundant

Correlations with P values in bold are statistically significant

**Supplementary table 5.** Correlations between analyte level changes in NDEVs from baseline to 16-week outcome

***Control group***

| | *Analyte* | | | | | | | | | | | |
|---|---|---|---|---|---|---|---|---|---|---|---|---|
| | *proBDNF* | | | | *BDNF* | | | | *Humanin* | | | |
| *Analyte* | *Spearman r* | *95% C.I.†* | *P value* | *Sample size* | *Spearman r* | *95% C.I.†* | *P value* | *Sample size* | *Spearman r* | *95% C.I.†* | *P value* | *Sample size* |
| BDNF | 0.35 | -0.01 to 0.63 | **0.04750** | 32 | - | - | - | - | - | - | - | - |
| Humanin | -0.01 | -0.41 to 0.39 | 0.94582 | 26 | 0.31 | -0.10 to 0.63 | 0.11886 | 26 | - | - | - | - |
| Irisin | 0.10 | -0.34 to 0.49 | 0.65661 | 24 | 0.46 | 0.07 to 0.73 | **0.01929** | 25 | 0.25 | -0.22 to 0.62 | 0.27312 | 21 |

***Exercise group***

| | *Analyte* | | | | | | | | | | | |
|---|---|---|---|---|---|---|---|---|---|---|---|---|
| | *proBDNF* | | | | *BDNF* | | | | *Humanin* | | | |
| *Analyte* | *Spearman r* | *95% C.I.†* | *P value* | *Sample size* | *Spearman r* | *95% C.I.†* | *P value* | *Sample size* | *Spearman r* | *95% C.I.†* | *P value* | *Sample size* |
| BDNF | 0.06 | -0.32 to 0.42 | 0.75509 | 30 | - | - | - | - | - | - | - | - |
| Humanin | 0.30 | -0.09 to 0.61 | 0.11723 | 28 | 0.03 | -0.37 to 0.41 | 0.88702 | 27 | - | - | - | - |
| Irisin | 0.01 | -0.44 to 0.47 | 0.95232 | 20 | 0.48 | 0.04 to 0.76 | **0.02843** | 21 | 0.26 | -0.27 to 0.67 | 0.31270 | 17 |

† Confidence interval

- redundant

Correlations with P values in bold are statistically significant
